# Numerical Investigation of Oxygenated and Deoxygenated Blood Flow through a Tapered Stenosed Arteries in Magnetic Field

**DOI:** 10.1371/journal.pone.0167393

**Published:** 2016-12-12

**Authors:** M. Y. Abdollahzadeh Jamalabadi, Amin Ali Akbari Bidokhti, Hamid Khak Rah, Siavash Vaezi, Payam Hooshmand

**Affiliations:** 1 Department of Mechanical, Robotics and Energy Engineering, Dongguk University, Seoul, Korea; 2 Department of Mechanical Engineering, Sharif University of Technology, Azadi St, Tehran, Iran; 3 Department of Mechanical Engineering, Shiraz Branch, Islamic Azad University, Shiraz, Iran; 4 Department of Mechanical Engineering, Sanandaj Branch, Islamic Azad University, Sanandaj, Iran; The Ohio State University, UNITED STATES

## Abstract

Current paper is focused on transient modeling of blood flow through a tapered stenosed arteries surrounded a by solenoid under the presence of heat transfer. The oxygenated and deoxygenated blood are considered here by the Newtonian and Non-Newtonian fluid (power law and Carreau-Yasuda) models. The governing equations of bio magnetic fluid flow for an incompressible, laminar, homogeneous, non-Newtonian are solved by finite volume method with SIMPLE algorithm for structured grid. Both magnetization and electric current source terms are well thought-out in momentum and energy equations. The effects of fluid viscosity model, Hartmann number, and magnetic number on wall shear stress, shearing stress at the stenosis throat and maximum temperature of the system are investigated and are optimized. The current study results are in agreement with some of the existing findings in the literature and are useful in thermal and mechanical design of spatially varying magnets to control the drug delivery and biomagnetic fluid flows through tapered arteries.

## 1- Introduction

The influence of magnetic field and metallic nanoparticles through the tapered stenosed arteries is interested for the purpose of drug delivery control [1]. The main criterion for mechanical design of that control is the surface tension on skin of the artery [2]. Mostly that effect is important when metachronal waves produced by large amount of particles act on walls [3–4] or peristaltic motion of wall [5–7]. In some psychological, body fluid processing and physical practice, bio-magnetic fluid dynamics through a sentoid tube in the presence of a magnetic field come about as a biofluid flows. Moreover, mechanical flow control devices has an interfering character that affect the fluid flow regime and make the stent break or embolism [8–10]. Effects of blood flow on decreasing the lumen of a blood vessel was shown by Mann et al. [8]. They demonstrate that the cardiovascular disease, for instance arteriosclerosis, is faithfully related to the fluid flow pattern in the blood vessels.

Many researchers were considered the effect of constant magnetic field on the blood flow [11], which behaves as a Newtonian or Non-Newtonian flow in order to understand the abnormal flow conditions of blood in a locally constricted blood vessel like a stenotic artery. When the multiphase blood is considered as a composition of red cells, white cells and platelets in plasma [12], the behavior of blood depends on vessel size. As the vessel size increase, the fluid can exhibit a Newtonian fluid manner, while at smaller arteries (~10 μm) the behavior changes to non-Newtonian with lower apparent viscosity [9]. In this condition, the effect of solid interaction of fluid is not ignorable as shown by Medhavi *et al*. [13].

The shape of stenosis is an important feature on the resistance to blood flow through an artery [14–18], which in combination with non-Newtonian effects leads to the complicate conditions [19,20] and bifurcations [21]. Transient transport of drug in a finite length tube could be disturbed by the fluctuations in fluid flow [22–24] due to heart beating (oscillatory pressure gradient in tapered arteries [10] and mild stenosis [9]) and presence of an external magnetic field [5,9] or combination of magnetic and electric field in a magneto hydrodynamic pump [11]. This interruption includes the movement of conductive physiological fluids. The transient condition could be aroused from periodic body acceleration [25] or stretching walls [26] that affect the shear‐thinning Non-Newtonian response [27–36]. Various fluid model [37–45] such as Carreau fluid model [37,38], Micropolar [39], power-law fluid [40], homogeneous Newtonian fluid [41–44], two-layered [45], Casson Fluid [46], Bingham plastic fluid [47], CNT nanofluid suspension [48,49], were used for the blood flow through artery in the presence of stenosis and strong static magnets [50] or spatially varying magnetic field [51–55].

As a brief search in literature, it seen that the numerical modeling of magnetic bio fluids under spatially varying magnetic field under the non-Newtonian assumption through a tapered stenosed arteries is not preformed yet. In this research, we investigated the effect of non-Newtonian behavior of blood flow on the resistance to flow, apparent viscosity, and wall shear stress in a stenosed artery by considering blood with a homogeneous Newtonian fluid, power-law fluid, and Carreau fluid model. Blood flow is considered through an axially non-symmetrical but radially symmetric stenosis where surrounded by a solenoid.

## 2- Mathematical Modeling

**Fig 1:**
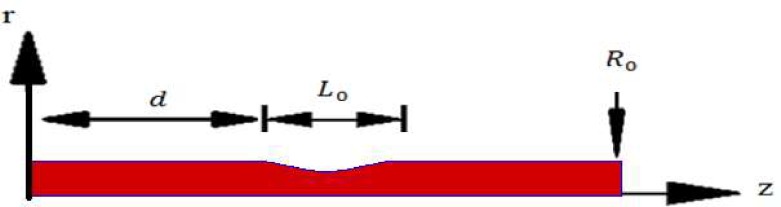
Schematic of problem consist of a stenoid artery in axisymmetric cylindrical coordinate system (r, z) with finite length.

As shown in Fig 1, a stenoid artery in axisymmetric cylindrical coordinate system (r,  z) with length L is considered in current problem which an incompressible (having a constant density ρ) flow of blood fluid. The distance of the artery wall from axis of the symmetry (r  =  0) is a function of axial position as:

$$\frac{R(z)}{R_{0}}=\left\{ \begin{array}{l} \;1;\;d<z\\ \;1-\frac{\delta }{2R_{0}}(1+cos\frac{2\pi }{L_{0}}(z-d-\frac{L_{0}}{2}));\;d<z\leq d+L_{0}\\ 1;\;L_{0}+d<z \end{array}\right.$$ (1)

The incompressible laminar flow of the system obeys the mass conservation equation and simplified as the equality of divergence of velocity vector to zero as:

$$\nabla \cdot v=\frac{\partial v_{r}}{\partial r}+\frac{v_{r}}{r}+\frac{\partial v_{z}}{\partial z}=0$$ (2)

As well the momentum equations in cylindrical coordinate are:

$$\rho \left(\frac{\partial v_{r}}{\partial t}+v_{r}\frac{\partial v_{r}}{\partial r}+v_{z}\frac{\partial v_{r}}{\partial z}\right)=-\frac{\partial p}{\partial r}+{\left(J\times B\right)}_{r}+\mu_{0}M\frac{\partial H}{\partial r}+\left[\frac{1}{r}\frac{\partial }{\partial r}\left(\mu r\frac{\partial v_{r}}{\partial r}\right)-\mu \frac{v_{r}}{r^{2}}+\frac{\partial^{2}v_{r}}{\partial z^{2}}\right]$$ (3)

$$\rho \left(\frac{\partial v_{z}}{\partial t}+v_{r}\frac{\partial z}{\partial r}+v_{z}\frac{\partial v_{z}}{\partial z}\right)=-\frac{\partial p}{\partial z}+{\left(J\times B\right)}_{z}+\mu_{0}M\frac{\partial H}{\partial z}+\mu \left[\frac{1}{r}\frac{\partial }{\partial r}\left(r\frac{\partial v_{z}}{\partial r}\right)+\frac{\partial^{2}v_{z}}{\partial z^{2}}\right]$$ (4)

where *μ*_0_ = 4*π* × 10^−7^ N/A^2^ is the permeability of free space. The viscosity for a Newtonian model of blood is:

$$\mu =10^{-3}$$ (5)

for a power law Non-Newtonian fluid is:

$$\mu \left(\dot{\gamma }\right)=0.017{\left(\dot{\gamma }\right)}^{-0.292}$$ (6)

and for a Carreau Non-Newtonian fluid is:

$$\mu \left(\dot{\gamma }\right)=0.036+0.02{\left(1+{\left(3.313\;\dot{\gamma }\right)}^{2}\right)}^{-0.3216}$$ (7)

The definition of auxiliary field is $\vec{Η}=\frac{1}{\mu_{0}}\vec{Β}-\vec{Μ}$ where

$$\vec{Η}=\frac{1}{\mu }\vec{Β}$$ (8)

**Fig 2:**
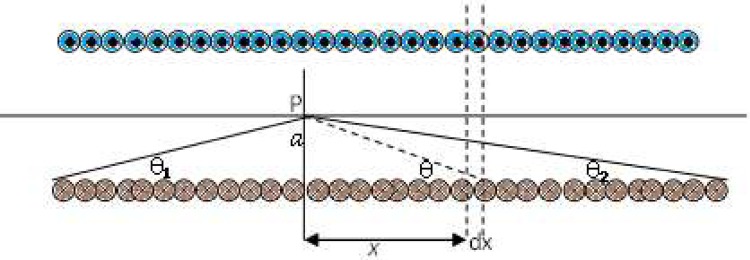
Schematic of the turns of the solenoid around the stenoid artery for the method of magnetic flux calculation.

The turns of the coil are exposed in Fig 2 with a dot demonstrating that the current is coming out of the page and a cross representative that it is going into the page. As shown in Fig 2, assume a point P in distance ‘a’ from the solenoid. By using the Biot Savart’s law in terms of the vector potential

$$\vec{B}\left(\vec{r}\right)=\frac{\mu_{0}}{4\pi }\int d^{3}r^{\prime }\frac{\vec{J}\left(\vec{r^{\prime }}\right)\times \left(\vec{r}-\vec{r^{\prime }}\right)}{{\left|\vec{r}-\vec{r^{\prime }}\right|}^{3}}=-\frac{\mu_{0}}{4\pi }\int d^{3}r^{\prime }\vec{J}\left(\vec{r^{\prime }}\right)\times \nabla \frac{1}{\left|\vec{r}-\vec{r^{\prime }}\right|}=\frac{\mu_{0}}{4\pi }\nabla \times \int d^{3}r^{\prime }\frac{\vec{J}\left(\vec{r^{\prime }}\right)}{\left|\vec{r}-\vec{r^{\prime }}\right|}$$ (9)

The calculation of the induced field is strait forward for a point on axis of solenoid as:

$$B={-\mu }_{0}nI\int_{{\pi -\theta }_{1}}^{\theta_{2}}\frac{\sec^{2}\;\theta \;\cot^{2}\;\theta d\theta }{2\;\cot^{3}\;\theta \;\sec^{3}\;\theta }=-\frac{\mu_{0}nI}{2}\int_{\pi {-\theta }_{1}}^{\theta_{2}}\sin\;\theta d\theta =\frac{\mu_{0}nI}{2}(\cos\;\theta_{2}+\cos\;\theta_{1})$$ (10)

where the amount of current per length is “ **I.n** “ whereas the length of the solenoid approaches infinity (**θ**_**1**_ = **θ**_**2**_ → **0**) it leads to **B** = **μ**_**0**_**nI**. The analytical solution of finite solenoid is the function of complete elliptic integrals of the first, second, and third kind.

Magnetic field equations are:

$$\nabla .B=\nabla .\mu_{0}\left(H+M\right)=\frac{\partial B_{r}}{\partial r}+\left(\frac{B_{r}}{r}+\frac{1}{r}\frac{\partial B_{\theta }}{\partial \theta }\right)+\frac{\partial uB_{z}}{\partial z}=0$$ (11)

and

J=σ(V×B) (12)

In our study, the presumed electrical conductivity of blood is uniform (0.8 Sm^-1^). Energy equation for the axisymmetric case of the system is derived as:

$$\rho C_{p}\frac{DT}{Dt}+\mu_{0}T\frac{\partial M}{\partial T}\frac{DH}{Dt}-\frac{J.J}{\sigma }=k\nabla^{2}T+2\mu \left[{\left(\frac{\partial u_{r}}{\partial r}\right)}^{2}+{\left(\frac{\partial u_{z}}{\partial z}\right)}^{2}+{\left(\frac{\partial u_{r}}{\partial z}+\frac{\partial u_{z}}{\partial r}\right)}^{2}\right]$$ (13)

In the above equation, H is for a linear media that has the following form:

$$\vec{Μ}=\chi \vec{Η}$$ (14)

Along the axis of symmetry, the normal component of the velocity, the axial temperature gradient, the axial velocity gradient and the shear stress vanish. These may be stated mathematically as the boundary conditions:

$$u_{r}\left(x,r=R\right)=u_{z}\left(x,r=R\right)=0$$ (15)

$$u_{r}\left(x,r=0\right)=0$$ (16)

$$p\left(x=0,r\right)=p_{m}\text{cos}\left(\omega t\right)$$ (17)

$$\frac{\partial T}{\partial r}\left(x,r=R\right)=h\left(T\left(x,r=R\right)-T_{\infty }\right)$$ (18)

$$\frac{\partial T}{\partial r}\left(x,r=0\right)=0$$ (19)

$$T\left(x=0,r\right)=T_{\infty }$$ (20)

We introduce the nondimensional variables

$$\begin{array}{l} \bar{r}=\frac{r}{R},\;\bar{z}=\frac{z}{R},\;{\bar{v}}_{r}=\frac{v_{r}R}{\nu },\;{\bar{v}}_{z}=\frac{v_{z}R}{\nu },\;\bar{p}=\frac{pR^{2}}{\rho \nu^{2}},\;\theta =\frac{T}{T_{\infty }},\\ \bar{t}=\frac{t\nu }{R^{2}},\;\bar{H}=\frac{H}{H_{\max}},\;Ha=\sqrt{\frac{\sigma }{\nu \rho }}R\mu_{0}H_{\max},\;Mn_{F}=\frac{\mu_{0}\chi }{\rho }{\left(\frac{RH_{\max}}{\nu }\right)}^{2}\\ \mathrm{Pr}=\frac{\rho C_{p}\nu }{k},\;Q_{V}=\frac{1}{C_{p}T_{\infty }}\frac{\nu^{2}}{R^{2}},\;Q_{J}=\frac{\sigma }{C_{p}\rho T_{\infty }}{\left(\frac{H_{\max}}{\mu_{0}}\right)}^{2} \end{array}$$ (21)

By substitution of the above relations (in Eq (21)) to the governing Eqs (2–4) and (13) the following non-dimensional system of partial differential equations is obtained for:

continuity

$$\frac{\partial {\bar{v}}_{r}}{\partial \bar{r}}+\frac{{\bar{v}}_{r}}{\bar{r}}+\frac{\partial {\bar{v}}_{\bar{z}}}{\partial \bar{z}}=0$$ (22)

z-momentum

$$\frac{\partial {\bar{v}}_{\bar{z}}}{\partial \bar{t}}+{\bar{v}}_{\bar{r}}\frac{\partial {\bar{v}}_{\bar{z}}}{\partial \bar{r}}+{\bar{v}}_{\bar{z}}\frac{\partial {\bar{v}}_{\bar{z}}}{\partial \bar{z}}=-\frac{\partial \bar{p}}{\partial \bar{z}}-{\left(Ha\bar{H}\right)}^{2}{\bar{v}}_{\bar{z}}+\frac{\partial^{2}{\bar{v}}_{\bar{z}}}{\partial {\bar{r}}^{2}}-\frac{{\bar{v}}_{\bar{z}}}{\bar{r}}+\frac{\partial^{2}{\bar{v}}_{\bar{z}}}{\partial {\bar{z}}^{2}}$$ (23)

r-momentum:

$$\frac{\partial {\bar{v}}_{\bar{r}}}{\partial \bar{t}}+{\bar{v}}_{\bar{r}}\frac{\partial {\bar{v}}_{\bar{r}}}{\partial \bar{r}}+{\bar{v}}_{\bar{z}}\frac{\partial {\bar{v}}_{\bar{r}}}{\partial \bar{z}}=-\frac{\partial \bar{p}}{\partial \bar{r}}-Mn_{F}\bar{H}\frac{\partial \bar{H}}{\partial \bar{z}}+\frac{\partial^{2}{\bar{v}}_{r}}{\partial {\bar{r}}^{2}}-\frac{{\bar{v}}_{r}}{\bar{r}}+\frac{\partial^{2}{\bar{v}}_{r}}{\partial {\bar{z}}^{2}}$$ (24)

energy:

$$\begin{array}{l} \frac{\partial \theta }{\partial \bar{t}}+{\bar{v}}_{\bar{r}}\frac{\partial \theta }{\partial \bar{r}}+{\bar{v}}_{\bar{z}}\frac{\partial \theta }{\partial \bar{z}}=\frac{1}{\mathrm{Pr}}\left(\frac{\partial^{2}\theta }{\partial {\bar{r}}^{2}}+\frac{\theta }{\bar{r}}+\frac{\partial^{2}\theta }{\partial {\bar{z}}^{2}}\right)+\\ Q_{V}\left(2{\left(\frac{\partial {\bar{v}}_{\bar{r}}}{\partial \bar{r}}\right)}^{2}+2{\left(\frac{{\bar{v}}_{\bar{r}}}{\bar{r}}\right)}^{2}+2{\left(\frac{\partial {\bar{v}}_{\bar{z}}}{\partial \bar{z}}\right)}^{2}+{\left(\frac{\partial {\bar{v}}_{r}}{\partial \bar{z}}+\frac{\partial {\bar{v}}_{\bar{z}}}{\partial \bar{r}}\right)}^{2}\right)+Q_{J}{\bar{H}}^{2}{\bar{v}}_{\bar{z}}{}^{2} \end{array}$$ (25)

Thermal heat transfer coefficient for temperature boundary conditions on the wall is calculated from the natural convection of horizontal tube. As made known, average blood vessels diameter in ascending aorta is 25 mm, descending aorta is 23.3 mm, abdominal aorta is 19.5 mm, femoral artery is 12.9mm, arterioles is 1.37 mm, and capillaries is 0.48 mm where the velocity is changed from 40 cm/s in aorta to 0.03 cm/s in capillaries with normal 1.2 beats per second. Many texts cite the normal resting adult human heart rate range from 60–100 bpm. Numerous studies, along with expert agreement show that the normal resting adult heart rate is possibly nearer to a range between 50–90 bpm. Throughout sleep a slow heartbeat with rates about 40–50 bpm is common and is considered normal.

**Fig 3:**
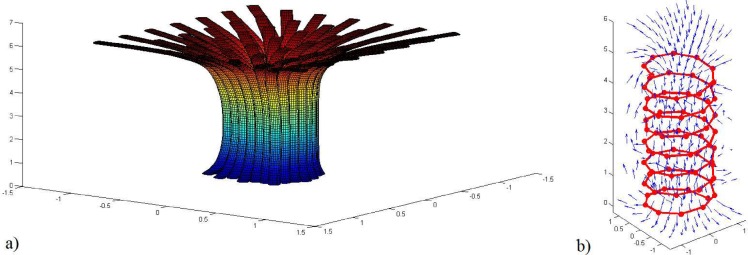
Distribution of magnetic flux in domain a) Stream tube, b) normalized vectors.

The stream tube of magnetic flux field is portrayed in Fig 3A for the solenoid configuration with 70 segments in 7.5 cycle of wire, which has the pitch angle of 1/10. In addition, the normalized vector plot of magnetic field based on Eqs (8–14) is revealed in Fig 3B. As it is apparent, the magnetic flux is approximately uniform inside the solenoid and completely symmetrical.

**Fig 4:**
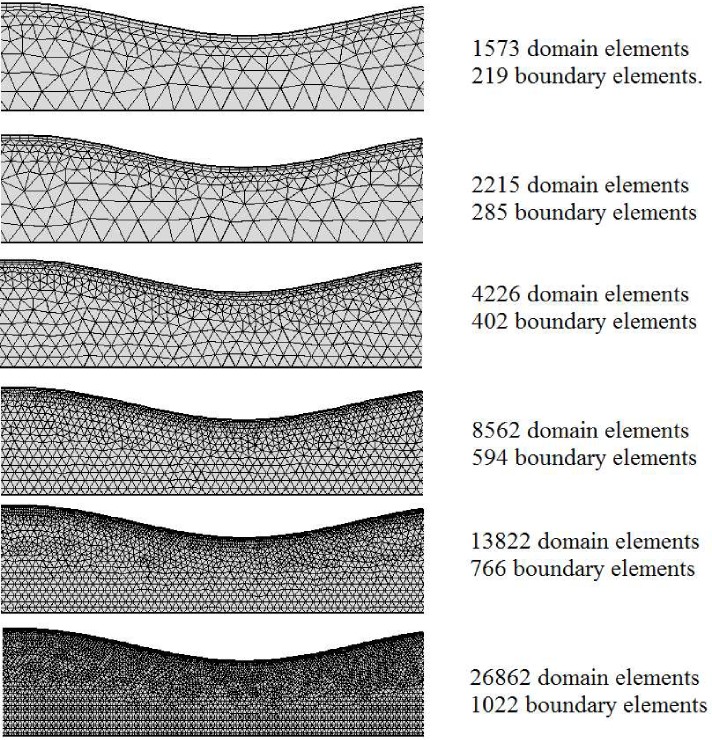
Mesh configurations for various cases.

Nonlinear governing Eqs of (22–25) are converted into computational domain and reduced to the second-order accuracy finite volume equations by SIMPLE algorithm of discretization. Then, the system of equations subject to the boundary conditions (15–21), is solved applying a Ronge-Kutta numerical technique. Meshed element of computational domain around the stent is depicted in Fig 4 for various mesh configurations. As shown complete mesh consists of 1573, 2215, 4226, 8562, 13822, 26862 respectively and 219, 285, 402, 594, 766, 1022 boundary elements respectively from top to the bottom. The error analysis of various mesh size is presented in Table 1 with velocity solution on mesh consists of 93001 domain elements and 1319 boundary elements.

**Table 1:** Error analysis of various mesh size.

Boundary elements	Total elements	Maximum Relative error on V_z_
219	1573	4.42%
285	2215	3.87%
402	4226	1.212%
594	8562	0.97%
766	13822	0.5%
1022	26862	0.1%

## 3- Results and Discussions

**Fig 5:**
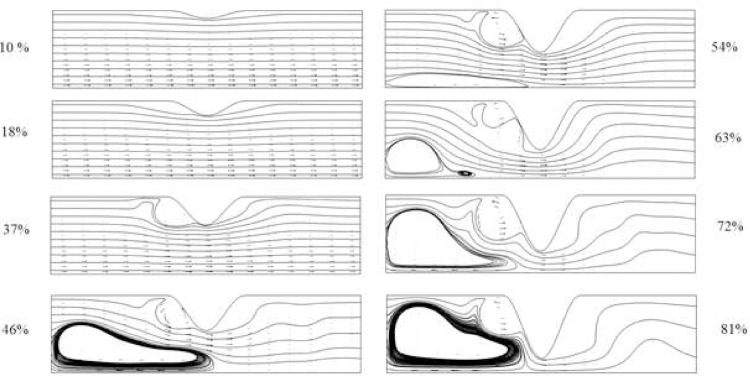
Velocity vectors and streamline at t = 1 for various blockage for Case 1 of Table 2.

In general, the velocity though the blood vessel and shear stress at the stenosis zone are of interest. The blood is under the influence of a solenoid magnetic field and a pulsatile blood flow in this research; consequently, the fluid thermal and flow characteristics is a function of time and position. Various cases considered in research with Hartmann number and corresponding magnetic numbers are presented in Table 2. In Fig 5 the distribution of velocity vectors and corresponding streamlines at dimensionless time, equals to unity for various blockages are demonstrated. As shown the two region of recirculation are existing in the flow field, which their size increases by increase of blockage percent. The first recirculation is appeared behind the stent while the second is appeared in for the stenosis ratio over than 46 percent of the blood vessel. The boundary layer proportionally grows in respect to the fluid flow outside it. The both zone increase while at stenosis ratio of 81 percent their boundaries touch each other. As well the wake expansion with time variation is obvious in the Fig 5.

**Table 2:** Hartmann number and corresponding magnetic numbers.

*Case no*	Ha	Mn_*F*_ Deoxygenated	Mn_*F*_ Oxygenated
1	0.5	2.8×10^5^	-5.3×10^4^
2	1	1.12×10^6^	-2.1×10^5^
3	2	4.49×10^6^	-8.43×10^5^
4	3	1.1×10^7^	-2.07×10^6^
5	4	1.8×10^7^	-3.4×10^6^

**Fig 6:**
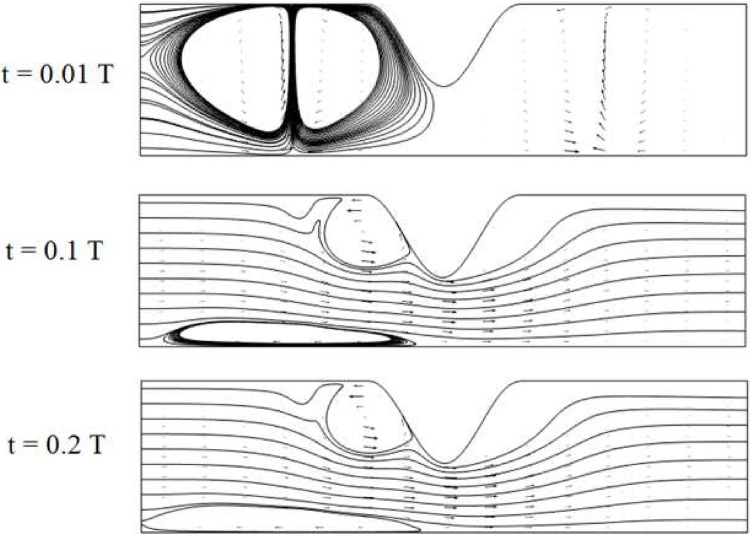
Velocity vectors and streamline at various times for 54 percent blockage for Case 1 of Table 2.

The time evolution of flow parameters and the flow pattern in a blood vessel in the presence of a stenosis is exposed in Fig 6 for 54 percent blockage for Case 1 of Table 2. As made known by the velocity vectors and streamline, at the dimensionless time equals to 0.01(T = R^2^/υ), two big recirculating zone are made in front of each other behind the stent between symmetry line and vessel solid wall. The boundary of these two cells is approximately in correspondence of the position of the beginning of the solenoids where the highest values of magnetic flux change are prevailing. As times evolves the fluid opens its way between this two cells and the cell near the vessel opening broke to the smaller cell as shown in dimensionless time of 0.1. After then, although the velocity and pressure through the vessel will change, but the streamline configuration remains unchanged.

**Fig 7:**
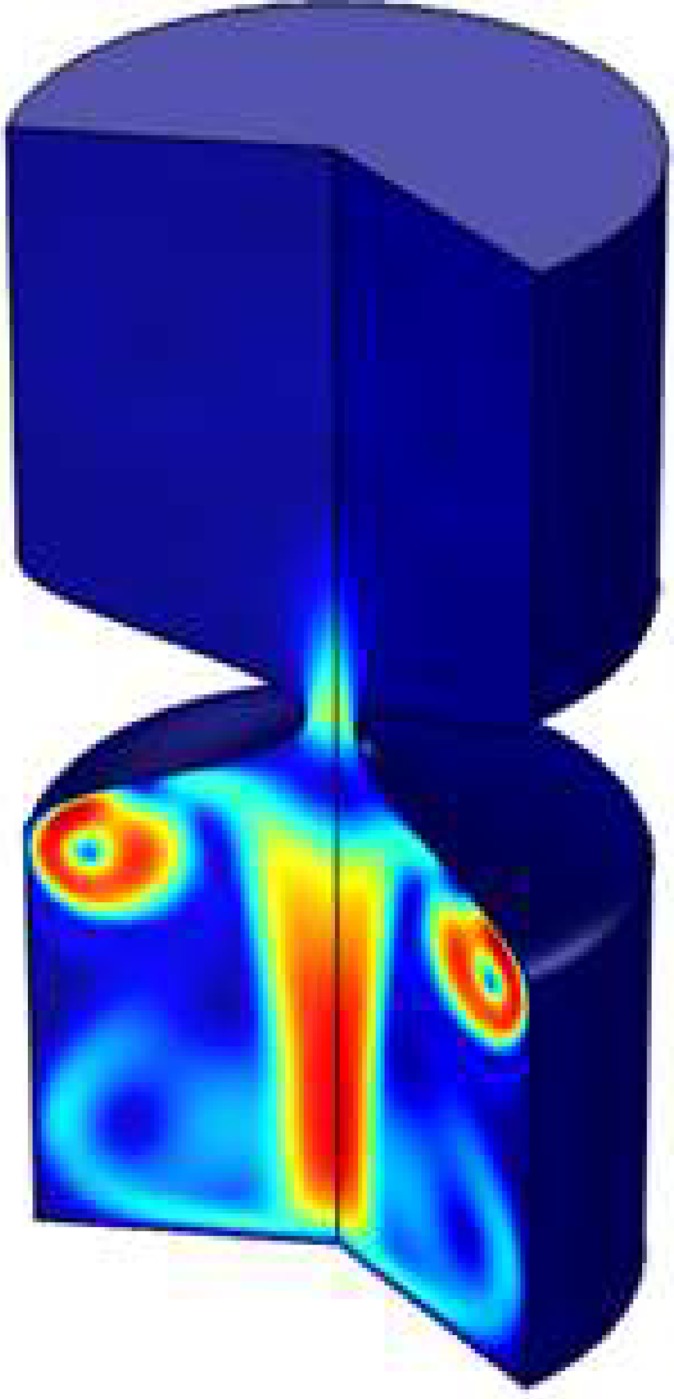
Velocity magnitude at t = 1 throat (90% blockage) for Case 1 of Table 2.

**Fig 8:**
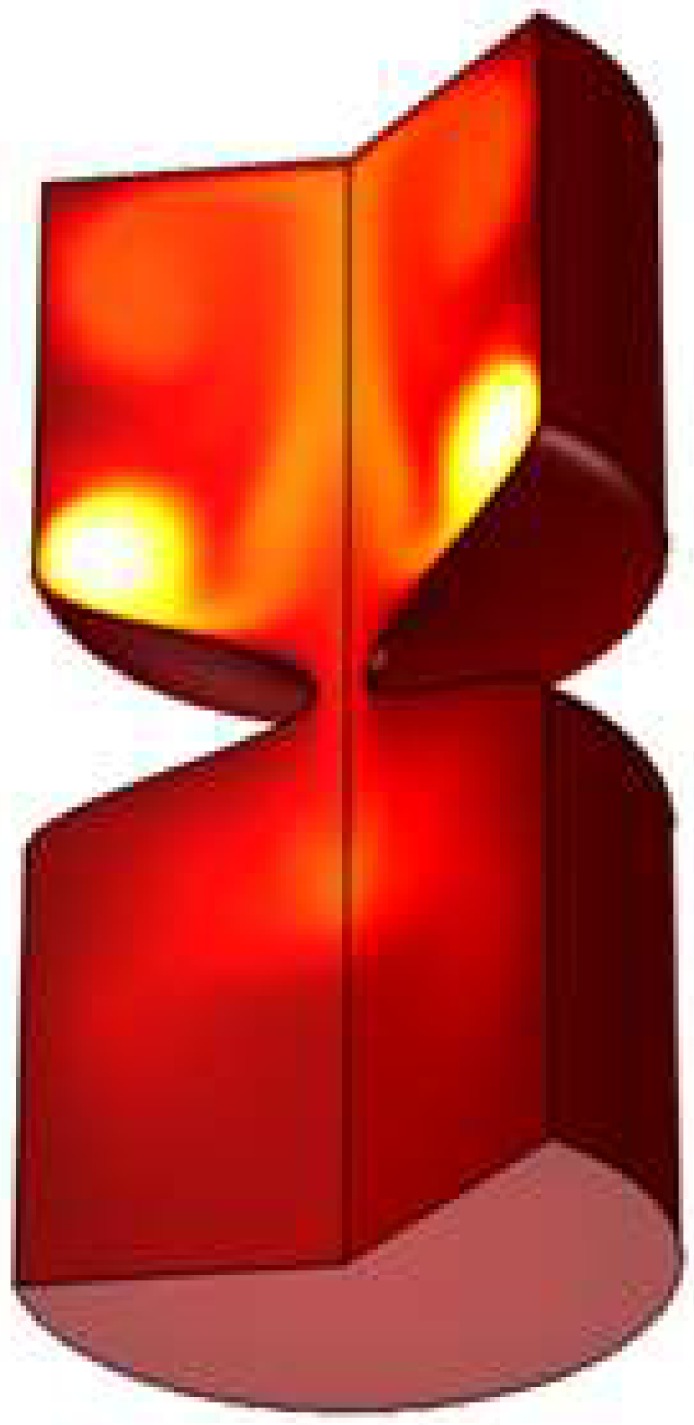
Temperature at t = 1 throat (90% blockage) for Case 1 of Table 2.

The maximum magnitude of the velocity and temperature is happened behind the stent as shown by Fig 7 and Fig 8 respectively. As shown at the dimensionless time equals to 0.01 for Case 1 of Table 2 at 90% blockage the highest value of thermal and kinetic energy happens before the throat.

**Fig 9:**
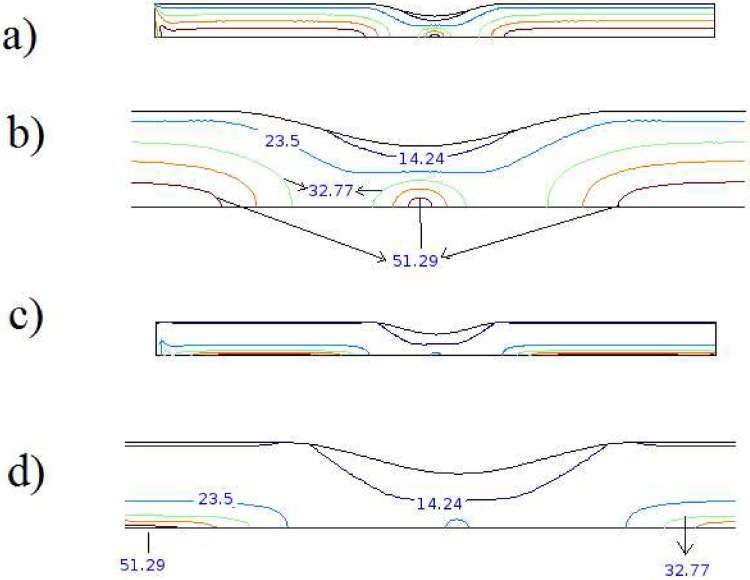
Contours of relative viscosity (relative to Newtonian viscosity) for 37% blockage at t = 1 for various Non-Newtonian fluid models a) Careau model in whole vessel b) Careau model near the throat c) power law model in whole vessel d) power law model near the throat.

The proper choose of fluid model is an important key rule to calculate flow field variables. As shown in the Fig 9 the relative viscosity of Power-law and Carreau methods are plotted to show the difference between models. As shown, because of high shear rates in this case, the Non-Newtonian calculated viscosity are one order of magnitude higher than the Newtonian case. The lowest Non-Newtonian viscosity (~O(10)) are exist in the wall of the vessel, while the higher values (~O(50)) happens on symmetry line between openings and the throat. As there is an inverse correlation among Non-Newtonian viscosity and velocity gradients, viscosity on the vessel wall distant from the stenosis is little and great viscosity is identified in the course of the axis of symmetry. As well, the vortex at the finale of the stenosis efforts away, the high viscosity units at this vortex region amounts to the walls of the arterial vessel and eventually change just beforehand the axis of symmetry.

**Fig 10:**
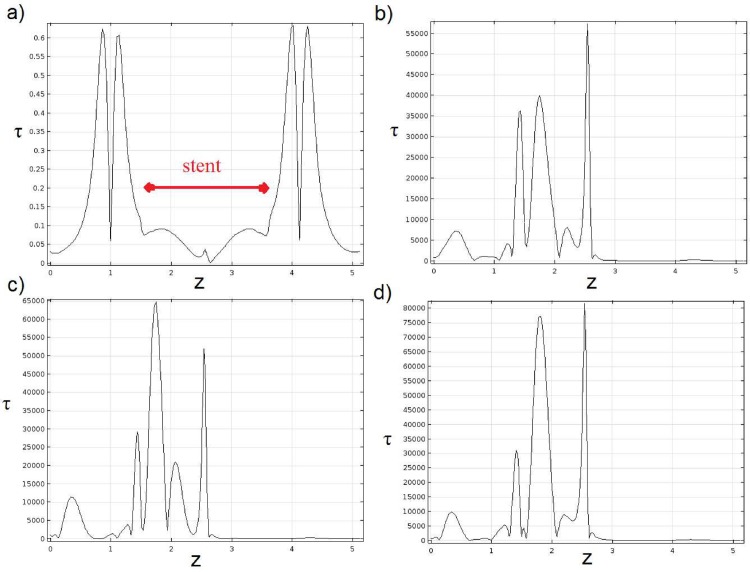
Dimensionless shear tension versus wall curve length for 30% stent ratio at various times: a) t = 0.01T, b) t = 0.1T, c) t = 0.2T and d) t = 0.3T.

Dimensionless plot of shear tension at t = 0.01T for 28% stent ratios is plotted in Fig 10A. As shown, the shear tension increase up to the first recirculation zone and decrease suddenly near the separation point before the stent because of the lower shear rates inside the recirculation section and high vorticities. At this region, we have an approximately smooth region and then a similar behavior of inlet opening will observe at the outlet opening. By time evolution at t = 0.1T a peak of shear tension is seen at throat while another local maximum is seen at the beginning of stent (See Fig 10B). After the stenosis peak, recirculation and stent shape are highly important as a change in shear tensions can exacerbate conditions and additional expand the stenosis. Dimensionless plot of shear tension for t = 0.2T and 0.3T are depicted in in Fig 10C and 10D. As shown the local maximums have closer values together and compete to reach a higher shear tension value.

**Fig 11:**
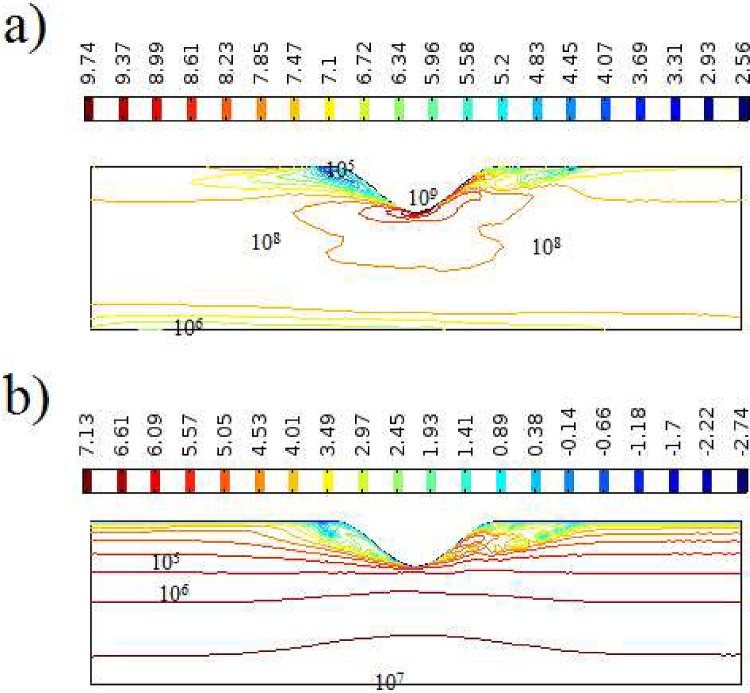
General configurations of heating sources for t = T and 28% stent ratio a) viscous heating Q_v_ = 1 b) Joule heating Q_J_ = 1.

The overall outline of viscous heating in current study for stent ratio of 0.28 and Q_v_ = 1 is plotted in Fig 11A. As shown the maximum volumetric heating rate happens at stent wall (10^9^) which remains constant throughout the tube (10^8^) and the lower values occurs near the symmetry line (10^6^), while the minimum come about in the recirculation zone after the stent (10^5^). As well, the global shape of Joule heating in current study for stent ratio of 0.3 and Q_J_ = 1 is plotted in Fig 11B. As shown the maximum volumetric Joule rate transpires about the symmetry line (10^7^). By increase of radial distance from the centerline, the value decreases while the minimum values achieved at the two-recirculation zones around the stent (10^1^). Granting the viscous heated blood temperature values in this Fig is higher than Joule heated values for unit coefficient of heating; in reality, the Q_v_ has the lower values, and the furthermost of the heating source is attributable to MHD heating. The upsurge in the temperature of blood is tremendously vital and an action to alleviate it is domineering. Even though an inline microwave blood warmer may be used to heat blood safely to 49 degrees C. Blood warmed to this temperature may significantly increase the amount of heat returned to the hypothermic trauma patient.

**Fig 12:**
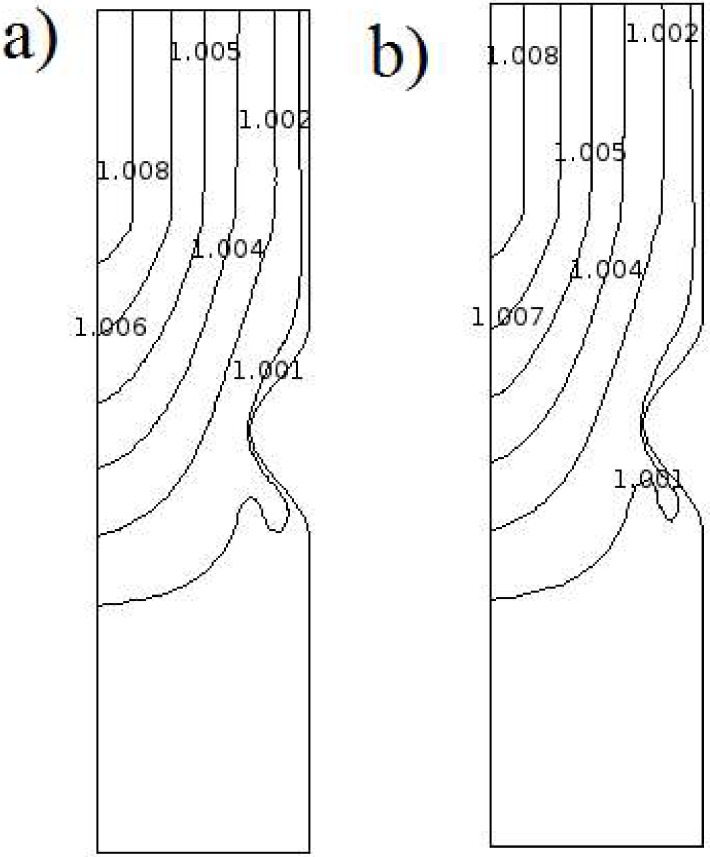
Temperature distribution for 28% stent ratio Q_v_ = 0.001 and Q_J_ = 1 a) t = 0.01T b) t = T.

Fig 12 presents the initial and final temperature distribution for 28% stent ratio low viscous heating Q_v_ = 0.001 and normal Joule heating Q_J_ = 1. The overall pattern of temperature contour which is obtained at small time after starting from initial condition (θ = 1) remains until the end of process. As given away, the temperature increase after the stent and maximum temperatures materializes at the symmetry line outlet. This location is besides the stenosis throat to be affected by stent. If the maximum obtained here normalized by the 37 Celsius degree of internal human body (rectal, vaginal, ear), the 2.4 increase in blood temperature is attained. An early morning temperature higher than 37.2°C (99.0°F) or a late afternoon temperature higher than 37.7°C (99.9°F) is normally considered a fever. Normally, the increase of blood temperature higher than 42 degrees Celsius can affects its physiological property, permanent harms to protein of plasma and fainting, dehydration, weakness, vomiting, headache, breathlessness and dizziness. As well, the temperatures 44°C (111.2°F) or more almost certainly death will occur; however, people have been known to survive up to 46.5°C (115.7°F).

**Fig 13:**
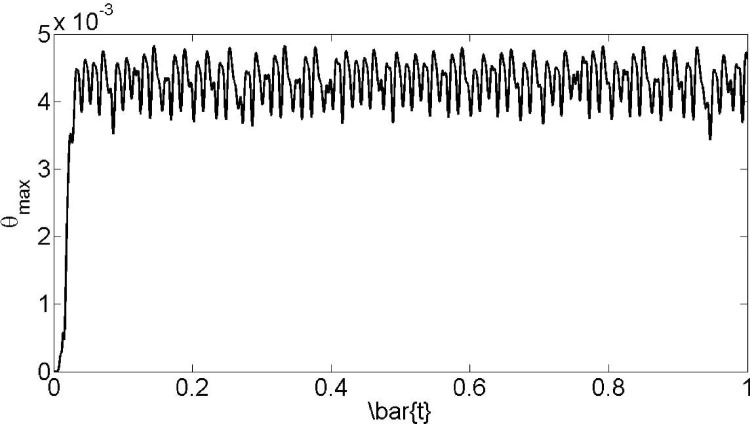
Maximum temperature versus time for 95% stent ratio.

Fig 13 presents the maximum temperature throughout domain versus time for 95% stent ratio. At the blood vessel, the chronological development of temperature field is a sudden increase to the quasi steady state condition with an small time constant and then fluctuates because of the pressure oscillation which flow experienced. The advection of velocity field affects the change of maximum temperature and its place. In the meantime, the imposed magnetic field and the pressure vacillations transformed temperature field.

**Fig 14:**
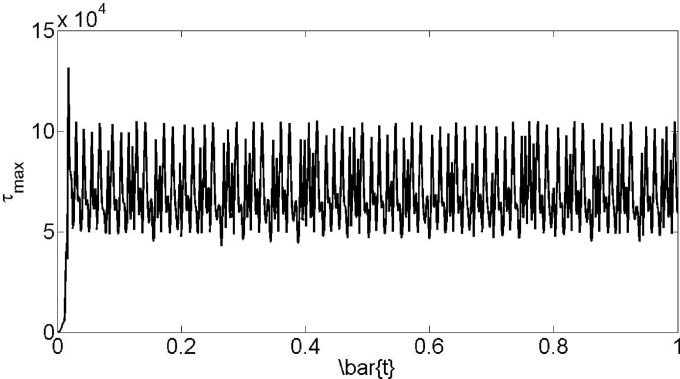
Maximum shear tension versus time for 95% stent ratio.

Fig 14 indicates the abovementioned occurrences for maximum shear tension versus time for 95% stent ratio, which shadowed the same tendency, happened for the temperature. In addition to this fact, it is obvious that the shear stress reached an extreme at blood vessel walls. The fluctuations are large and the maximum is approximately two times of the minimum values.

**Fig 15:**
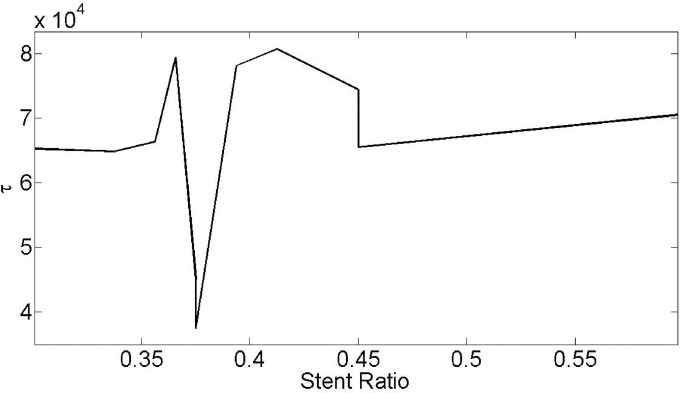
Finding optimal stent ratio with minimum shear tension in Case 1 of Table 2.

Fig 15 finding optimal stent ratio with minimum shear tension in Case 1 of Table 2. As extreme shear tension alteration through the time domain, this minimization is also on all position and time space. Through the optimum search from stent ratio from 0.01 to 0.99, the value of 0.375 finally adopted. Bi-conjugate gradient stabilized method is used to discovery the optimal stent ratio with minimum shear tension (See Supporting Information of Fig 15).

**Fig 16:**
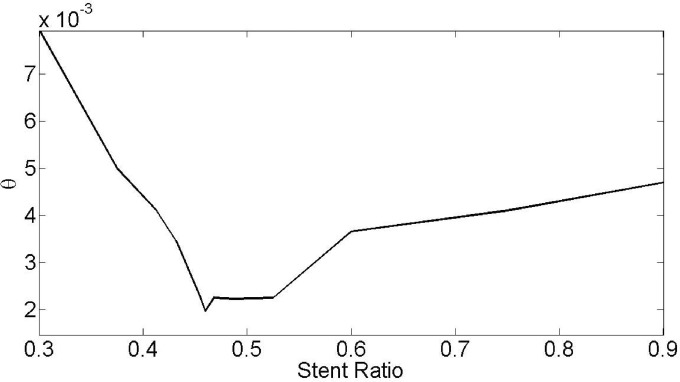
Finding optimal stent ratio with minimum temperature in Case 1 of Table 2.

Fig 16 finding optimal stent ratios with minimum temperature in Case 1 of Table 2. The variation in temperature is due to velocity field and boundary heat convection inside the blood vessel. The use of Bi-conjugate gradient stabilized method in the range of stent ratio from 0.01 to 0.99, the value of 0.45938 finally approved (See Supporting Information of Fig 16).

**Fig 17:**
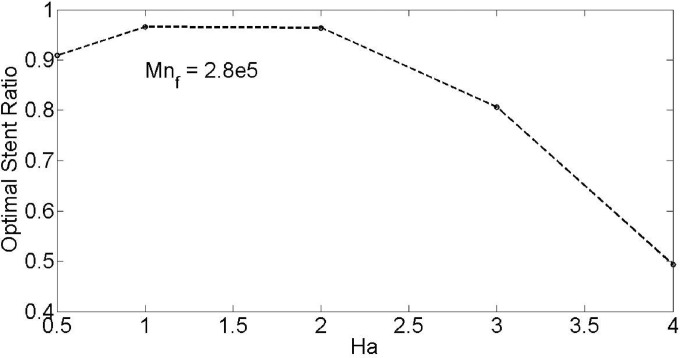
Effect of Hartmann number on optimal stent ratio with minimum temperature.

With the purpose of study the effect of magnetic field and its space change on the optimal stenosis shape, we examined the effect of Hartmann number and Magnetic number on the minimum temperature all over the of blood vessel. Fig 17 illustrates the effect of Hartmann number on optimal stent ratio with minimum temperature. As publicized, by increase of Hartmann number higher than unity, the optimal stent ratio decreases while the behavior is diverse for values less than unity (See Supporting Information of Fig 17).

**Fig 18:**
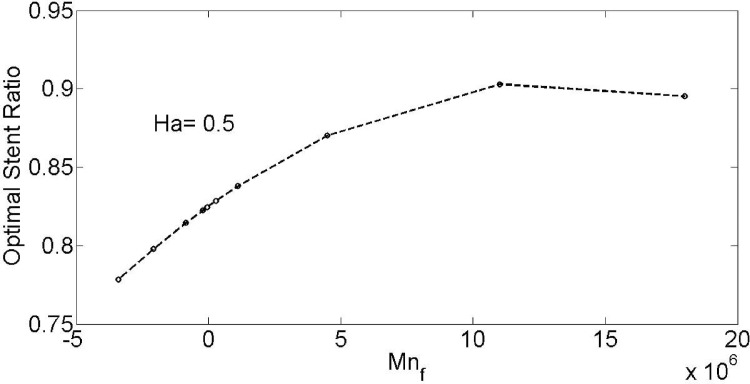
Effect of Magnetic number on optimal stent ratio with minimum temperature.

Fig 18 illustrates the effect of Magnetic number on optimal stent ratio with minimum temperature. As shown by increase of Magnetic number more blockages are needed for lessen the maximum temperature. As shown based on the value of Magnetic number oxygenated and deoxygenated blood flow through a tapered stenosed arteries in magnetic field can be affected the optimal stent ratio for the temperature minimization (See Supporting Information of Fig 18).

## 4- Conclusions

A transient computational tool is developed to study the Newtonian and non-Newtonian fluid model of biomagnetic blood through a tapered stenosed arteries surrounded a by solenoid under the presence of heat transfer. The code was based on the SIMPLE algorithm for structured grid. The effects of fluid viscosity model, Hartmann number, and magnetic number on wall shear stress, shearing stress at the stenosis throat and maximum temperature of the system are investigated and are optimized and the following consequences are found:

- By the use of solenoid around the tapered stenosed arteries contains biomagnetic flow, the main characteristics of fluid flow and thermal aspects can be controlled.
- The non-Newtonian pulsatile flow reaches a stable fluid flow configuration soon from the start of motion.
- By use of external magnetic field the recirculation zone, vortex strength, and separated region can affected.
- In the attendance of stenosis, the wall shear stress is extremum on throat and after stent region.
- The viscous heating can affect the vessel walls while the Joule heating happens at the centerline of the vessel.
- The heat transfer through this pulsatile flow reaches a stable arrangement soon from the start of motion.
- The maximum temperature of blood and the maximum shear stress through the vessel are fluctuating versus time by the rate of pressure oscillations.
- Optimal stent ratio is found based on the applied magnetic field on biomagnetic blood flow.

## Supporting Information

S1 Fig Supporting Information of Fig 15. Finding optimal stent ratio with minimum shear tension in Case 1 of Table 2
(XLSX)

S2 Fig Supporting Information of Fig 16. Finding optimal stent ratio with minimum temperature in Case 1 of Table 2
(XLSX)

S3 Fig Supporting Information of Fig 17. Effect of Hartmann number on optimal stent ratio with minimum temperature
(XLSX)

S4 Fig Supporting Information of Fig 18. Effect of Magnetic number on optimal stent ratio with minimum temperature
(XLSX)

## Symbol Description unit

B: magnetic induction Tesla (= 10000 gauss)
C_p_: specific heat at constant pressure J/kg.K
F: body forces per unit volume N
H: magnetic field intensity
k: coefficient of thermal conductivity of the fluid W/m.K
J: density of the electric current
M: magnetization
p: pressure pa
T: temperature K
t: time m
V: blood velocity m/s

**Greek symbols**
α: thermal diffusivity m^2^/s
θ: dimensionless temperature, = T/T∞
σ: electrical conductivity of the fluid
μ: dynamical viscosity kg/m.s
υ: kinematic viscosity m^2^/s
ρ: fluid density kg/m^3^
χ: magnetic susceptibility

**Superscript**
max: Maximum
∞: ambient value
